# Spatial distribution of zero-dose children in Ethiopia: evidence for a targeted intervention from a large-scale cross-sectional evaluation survey

**DOI:** 10.3389/fped.2024.1337922

**Published:** 2024-04-04

**Authors:** Fisseha Shiferie, Samson Gebremedhin, Gashaw Andargie, Frank DelPizzo, Kidist Belete, Teferi Gedif Fenta

**Affiliations:** ^1^ Project HOPE Ethiopia Country Office, Addis Ababa, Ethiopia; ^2^ School of Pharmacy, Addis Ababa University, Addis Ababa, Ethiopia; ^3^ School of Public Health, Addis Ababa University, Addis Ababa, Ethiopia; ^4^ Bill & Melinda Gates Foundation, Seattle, WA, United States; ^5^ USAID Ethiopia, Addis Ababa, Ethiopia

**Keywords:** spatial analysis, zero-dose children, hotspot analysis, spatial interpolation, autocorrelation

## Abstract

**Background:**

Ethiopia is the fourth leading contributor to the global total of zero-dose children (those who lack the first dose of diphtheria-tetanus-pertussis containing vaccine) and has substantial regional variations in zero-dose children. This study explored the spatial pattern of zero-dose children aged 12–35 months in Ethiopia.

**Methods:**

A survey was conducted in pastoralist regions, developing regions, newly-established regions, conflict-affected areas, underserved urban populations, hard-to-reach areas, internally displaced populations, and refugees. Spatial autocorrelation was measured using the Global Moran's*I*statistic. Getis-Ord Gi* statistics was applied to calculate the spatial variability of the high and low prevalence rates of zero-dose children. The spatial interpolation technique was also applied to estimate unknown values that fall between known values. Inverse distance weighting interpolation method was used to predict the risk of zero-dose children. ArcGIS version 10.8 was used for the spatial analysis.

**Results:**

A total of 3,646 children aged 12–35 months were included in the study. The spatial distribution of zero-dose children in Ethiopia was non-random (Global Moran's*I* = 0.178971, *p* < 0.001). According to the hotspot analysis, western, eastern and northern parts of Somali and western and central parts of Afar regions had the highest load of zero-dose children (hotspot areas) followed by the Northeastern part of Amhara and southeastern part of Oromia regions. On the other hand, Southern Nations, Nationalities, and Peoples, Sidama, and the Eastern part of the Southwest Ethiopia peoples regions were identified as cold spot areas. The spatial interpolation analysis corresponded with the hotspot analysis results where western and central parts of Afar and western, eastern and northern parts of Somali regions were identified as high-risk areas for zero-dose children. However, Addis Ababa, Dire Dawa, Harari, Southern Nations, Nationalities, and Peoples, Sidama, Southwest Ethiopia Peoples, and parts of Oromia were found to be low-risk areas for zero-dose children.

**Conclusion:**

The spatial analysis identified that zero-dose children had a significant spatial variation across the study areas. High clusters of zero-dose children were detected in Afar and Somali regions. Implementing routine and mop-up vaccination campaigns in the identified hotspot areas will help Ethiopia to improve coverage and reduce immunization inequalities.

## 1 Introduction

Globally, the number of zero-dose children improved from 18.1 million in 2021 to 14.3 million in 2022 (1). Zero-dose children are those who lack the first dose of diphtheria-tetanus-pertussis containing vaccine (DTP1) (2). Almost all zero-dose children live in low-and middle-income countries, especially in African and Southeast Asian regions. Despite significant progresses made in increasing the number of immunized infants, Ethiopia is still the fourth leading contributor to the global total of zero-dose children with 1.1 million zero-dose children (3).

Spatial heterogeneity of vaccination coverage can delay disease elimination, even in countries with high national vaccination coverage rates (4, 5). Spatial analysis is a statistical method used to identify spatial clusters of events to recognize areas of greater vulnerability to health hazards (6). Exploring spatial heterogeneity in childhood vaccination is gaining attention to identify immunization gaps and intervene accordingly (7). Furthermore, understanding the variation in vaccination coverage is crucial for evidence-based decision-making in the prevention and control of vaccine-preventable diseases. Detecting spatial heterogeneity is useful in indicating existing programmatic gaps affecting progress that could not be identified through the routine monitoring of vaccination coverage (8).

In different sub-Saharan African countries, there is variation with regard to vaccination coverage which leads to weakened herd immunity and unequal disease risk (9). Although no study has mapped the geographical distribution of DTP-containing vaccine alone in Ethiopia, studies have demonstrated substantial regional variations in vaccination coverage of other vaccines. According to these studies, the lowest proportion of childhood immunization was in Somali, Afar, Northwest Gambella, Western and Eastern parts of Southern Nations, Nationalities, and Peoples (SNNP) and Oromia regions and the highest in Addis Ababa and Amhara region (10–14). Southwest Afar, East and Northwest Somali were at risk of Bacillus Calmette–Guérin (BCG) vaccination coverage (15). However, high BCG vaccination coverage was observed in northern, western, and central parts of Ethiopia (16). This could be because these regions are border areas and, hence, could not access and utilize healthcare services. The lower educational status of the people who live in these regions may also contribute to the low proportion of childhood vaccination (17). Local clusters of areas with low childhood measles-containing vaccine coverage were detected in the Somali, Afar, Gambella, and Oromia regions of Ethiopia (18).

Identifying geographical variations in zero-dose children is critical to effectively prioritize and design targeted prevention and intervention programs to improve Ethiopia's national vaccination coverage. To our knowledge, the spatial distribution of zero-dose children has not yet been mapped in Ethiopia. Therefore, this study aimed to explore the spatial pattern of zero-dose children aged 12–35 months in Ethiopia.

## 2 Methods

### 2.1 Study design and settings

This research was a component of a cross-sectional assessment survey that was carried out in 2022 between May and July. A single round, cross-sectional survey design was used for the study's implementation.

In line with the understanding that nearly half of zero-dose and under-immunized children in low-income countries are from hard-to-reach communities, conflict-affected settings, or disadvantaged urban areas (19) and based on our analysis of secondary data sources that was done prior to the cross-sectional survey, this study targeted populations in the following eight partly overlapping settings.

1. Pastoralist regions and populations: Afar and Somali regions and specific pastoralist or semi-pastoralist settings in Oromia, SNNP, Southwest Ethiopia Peoples, and Gambella regions
2. Developing regions: Afar, Somali, Gambella, and Benishangul Gumuz regions
3. Newly-established regions: Sidama and Southwest Ethiopia Peoples regions
4. Conflict-affected areas: Selected settings in Afar, Amhara, Oromia, and Benishangul Gumuz regions
5. Underserved urban populations: Urban slums in six selected cities (Addis Ababa, Bahirdar, Hawassa, Dire Dawa, Harar, and Adama) and rural areas under Dire Dawa City administration and Harari region
6. Hard-to-reach areas in major regions: Selected remote districts in Amhara, Oromia, and SNNP regions
7. Internally displaced populations (IDPs): Selected IDP centers in Afar, Amhara, Oromia, and Benishangul Gumuz regions
8. Refugees: Refugees from selected camps in Somali, Afar, and Gambella regions (20, 21, 22)

### 2.2 Study participants

Study participants included all children between the ages of 12 and 35 months who lived in underserved, remote, and conflict-affected areas of Ethiopia (20–22).

### 2.3 Sample size determination

The World Health Organization's (WHO) 2018 Vaccination Coverage Cluster Surveys manual served as the model for the study's sampling design (23). Adequate sample size for each of the target population was calculated using Cochran's Single Proportion Sample Size Formula (24) assuming 95% confidence level, 4% margin of error, 16% prevalence of zero-dose children (25), and 10% compensation for possible non-response. The following sample size formula was used to calculate the number of children required in a prevalence study: n=Z^2p(1−p)d^2 where *n* is the total sample size needed, *Z* is the statistic corresponding to 95% confidence level which is 1.96, *p* is the prevalence of zero-dose children in previous studies in Ethiopia, and *d* is precision (corresponding to effect size) (26). Therefore, a sample size of 360 was required for each of the aforementioned target population domains (20–22).

According to the Ethiopia Demographic and Health Survey (EDHS) 2016 and Mini EDHS 2019 data, an average of 12 children between the ages of 12 and 35 months are available per enumeration area (EA). An EA is a geographic area that is surveyed by one or more census representatives. To include as many eligible children as possible per EA, the 12- to 35-month age range was selected (25, 27). Therefore, assuming all children in the EAs would be eligible for inclusion in the study, at least 30 EAs were needed to recruit 360 children for each target population domain. In urban slums, 40 EAs were randomly selected and 480 children were drawn (Table 1) (20–22).

**Table 1:** Total sample size and EAs required for the vaccination coverage survey, Ethiopia, 2022.

Types of study population	Number of EAs	Total sample size
Afar	30	360
Somali	30	360
Benishangul Gumuz	30	360
Gambella	30	360
Pastoralist areas in Oromia, SNNP, and Southwest Ethiopia Peoples regions	30	360
Hard-to-reach areas	30	360
Conflict-affected areas	30	360
Refugees	30	360
IDPs	30	360
Newly-formed regions: Sidama and Southwest Ethiopia Peoples regions	30	360
Urban slums	40	480
Total	340	4,080

It was originally planned to include 4,080 children from 340 EAs (a minimum of 360 sample per population domain) in the survey. However, conflicts in some study districts led to the enrollment of 3,646 children aged 12–35 months from 340 EAs in the actual survey. The sample size was sufficiently large to allow subgroup analysis based on sex, age and other pertinent background characteristics such as socioeconomic status (20–22).

### 2.4 Sampling procedure

Using a cluster sampling technique, children between the ages of 12 and 35 months were selected in two steps (20, 21, 22). First, the entire pool of EAs in each target population domain was used to randomly select EAs. The EAs defined by the Central Statistical Agency of Ethiopia for the most recent census served as a sampling frame (25). Regarding urban slums, skilled cartographers identified, delineated and created EA maps for hotspot urban slums in Addis Ababa, Adama, Bahir Dar, Hawassa, Harar, and Dire Dawa. Villages or clusters were regarded as EAs in the context of IDPs and refugee camps. Second, a smartphone-based random number generator was used to select 12 children from a list of all eligible children in each EA (20–22).

### 2.5 Data collection procedures and data quality assurance

Pre-tested tools prepared in Amharic, Afan Oromo, Somali, Afar, and Sidama languages were used to collect the data. The CommCare digital app version 2.53.1 (Dimagi), an open-source and user-friendly application system that is interoperable with popular data analytics and visualization software (28), was used by 48 experienced enumerators and 24 supervisors to collect the survey data. The CommCare app helped to collect individual child-level and household information to ensure high-quality data collection, cleaning, and monitoring in real time. The digital app has also built-in features to support the global positions system data collection at the field level (20–22).

The recruitment of enumerators and supervisors was based on their educational background (at least diploma holders in a health-related field), familiarity with the CommCare digital app and previous experience in similar national surveys. A 5-day training guided by a structured training manual was provided to the enumerators and supervisors prior to deployment. An explanation of the sampling techniques, fundamentals of data collection, line-by-line discussion on the questionnaire, a synopsis of utilizing the CommCare digital app, mock interviews, field practice, and a review of basic ethical practices of research involving human subjects were all covered in the training (20–22).

Data collectors were allowed to collect data from up to six individuals per person per day. The supervisors re-interviewed one third of all the study participants to validate the quality of the data. Over the duration of the survey's implementation, the uploaded data were closely monitored by the research team (20–22).

### 2.6 Ascertainment of childhood vaccination

The vaccination status of each child was determined based on three distinct sources of information, as recommended by the WHO: vaccination cards, reports from the caregiver, and reports from the facility. Vaccination card was used to determine the child's immunization status in areas where the mother or caregiver had shown one. In situations when a vaccination card was unavailable, the mother's or caregiver's self-reports and recalls were used to determine the immunization status. The reliability of this approach to determine childhood vaccination in resource-constrained environments with inadequate childhood immunization records has been demonstrated by prior research (29).

### 2.7 Data management and spatial analysis

The data was stored in a local server on a daily basis. The descriptive and summary statistics, such as cross-tabulations and frequency tables, were generated using STATA version 17.0 (StataCorp, College Station, TX) (30). ArcGIS version 10.8 (Esri, California) was used for the spatial analysis (31). The data were weighted to make the survey representative. In order to balance weighted and unweighted sample size, linearization of post-stratification weights was made.

#### 2.7.1 Zero-dose children

The zero-dose children are those who lack the first dose of DTP1 (2).

#### 2.7.2 Spatial autocorrelation

In this study, spatial autocorrelation indicated whether the distribution pattern of zero-dose children aged 12–35 months across the study area was dispersed, clustered, or randomly distributed. The Global Moran's *I* spatial statistics was used to measure spatial autocorrelation by taking the entire data set. Moran's *I* is a statistic that produces a single output number between −1 and +1, and, for this study, a Moran's *I* value approaching +1 indicated that zero-dose children were spatially clustered. A Moran's *I* value approaching −1 indicated a dispersed spatial distribution of zero-dose children and a Moran's *I* value of 0 indicated a random geographic distribution of zero-dose children. A statistically significant Moran's *I* test confirmed the presence of a significant spatial autocorrelation (*p* < 0.05) and led to the rejection of the null hypothesis (zero-dose children were randomly distributed).

#### 2.7.3 Hotspot analysis (Getis-Ord Gi* statistics)

The spatial variability of the high and low prevalence rates of zero-dose children among children aged 12–35 months was calculated using Getis-Ord Gi* statistics in a hotspot analysis. The statistical significance of clustering was confirmed using the *Z*-score with a 95% confidence interval and a *p*-value <0.05. Statistical output with a high Gi* signifies zero-dose children hotspots (i.e., larger numbers of zero-dose children), while a low Gi* indicates zero-dose children cold spots (i.e., lower numbers of zero-dose children).

#### 2.7.4 Spatial interpolation

The spatial interpolation technique was applied to predict unknown values that fall between known values. Inverse distance weighting interpolation method was used to predict the risk of zero-dose children.

### 2.8 Ethical approval

The research was implemented in compliance with national and international ethical principles. The research protocol was reviewed and approved by the institutional review board of the Ethiopian Public Health Institute (416/2021). Information was collected after obtaining written informed consent from the caretakers. To maximize beneficence, all zero-dose children were referred to the nearest health facility using a referral form.

## 3 Results

### 3.1 Sociodemographic characteristics

The study included 3,646 mothers/caregivers who had children between the ages of 12 and 35 months. The response rate for the study was 97.7%. Of the respondents, 54% were between the ages of 25 and 34, and 59.2% had not received any formal education. Over 81% of those surveyed were from rural areas, and 17% were from the Afar region. At the time of the survey, 57% of respondents were unemployed, and over 90% of respondents were married or living with their partner (Table 2) (21).

**Table 2:** Sociodemographic characteristics of respondents in underserved settings of Ethiopia, 2022.

Characteristics	Frequency (*n* = 3,646)	Percent
Child's sex		
Boy	1,985	54.4
Girl	1,661	45.6
Child's age (months)		
12–23	1,849	50.7
24–35	1,797	49.3
Respondent's age (years)		
15–24	875	24.0
25–34	1,969	54.0
35–44	572	15.7
45 or above	105	2.9
Do not know	126	3.5
Respondent's educational status		
No formal education or preschool	2,158	59.2
Primary education	788	21.6
Secondary education	616	16.9
Tertiary education	84	2.3
Marital status		
Not ever married	43	1.2
Married/living together	3,312	90.8
Separated	83	2.3
Divorced	110	3.0
Widowed	98	2.7
Place of residence		
Urban	677	18.6
Rural	2,969	81.4
Caregiver's employment status		
Unemployed	2,098	57.6
Employed	1,548	42.4
Region^a^		
Afar	636	17.4
Amhara	372	10.2
Oromia	431	11.8
Somali	480	13.2
Benishangul Gumuz	216	5.9
SNNP	300	8.2
Sidama	239	6.6
Southwest Ethiopia Peoples	181	5.0
Gambella	479	13.1
Harari	60	1.6
Addis Ababa	192	5.3
Dire Dawa	60	1.6
Household size		
2–5	2,044	56.0
6 or above	1,602	44.0

^a^ Unweighted sample size.

### 3.2 Spatial autocorrelation analysis

**Figure 1:**
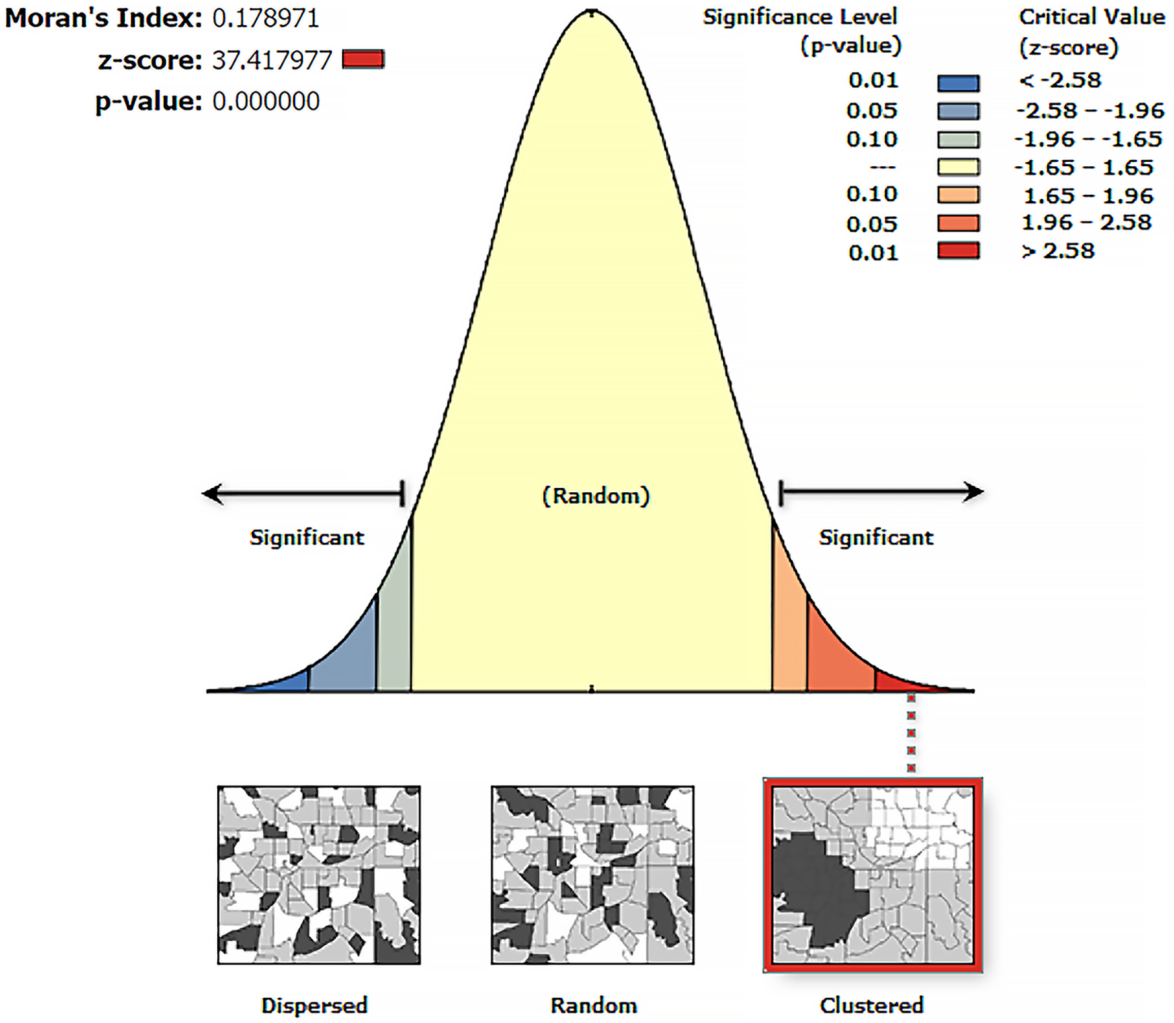
Spatial autocorrelation analysis of zero-dose children in Ethiopia, a cross-sectional evaluation survey.

The spatial autocorrelation analysis revealed that the spatial distribution of zero-dose children aged 12–35 months in Ethiopia was non-random (Global Moran's *I* = 0.178971, *p* < 0.001). The result showed that the observed Global Moran's *I* value was greater than the expected index (−0.000046) and the *p*-value was <0.01, which was statistically significant. The *Z*-score of 37.417977 indicated that there was a less than 1% likelihood that this clustered pattern could be the result of random chance (Figure 1).

### 3.3 Hotspot analysis of zero-dose children

**Figure 2:**
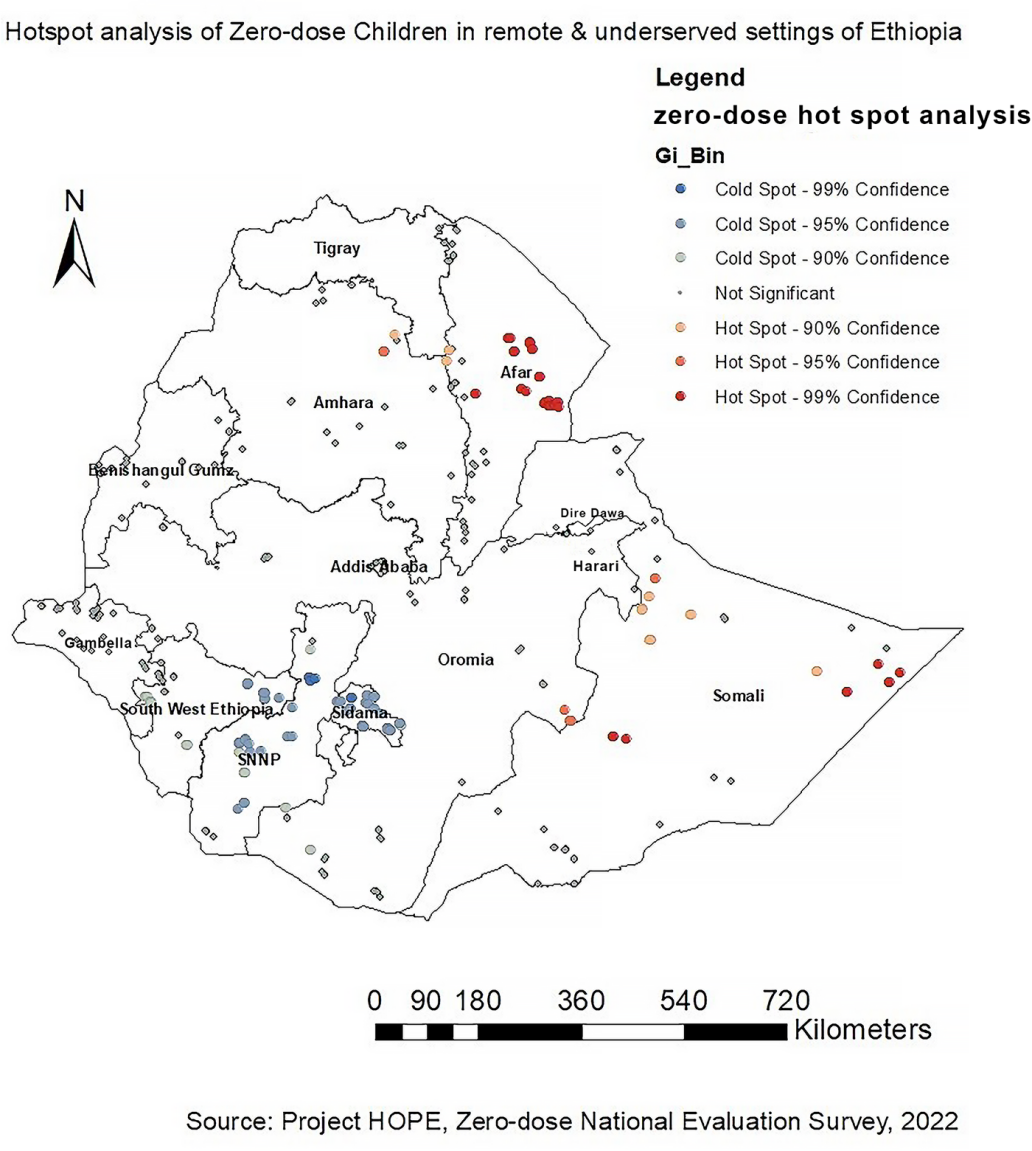
Hotspot analysis of zero-dose children in Ethiopia, a cross-sectional evaluation survey.

The dark red color indicates significant (*p* < 0.001) clusters of high zero-dose children (risk areas), whereas the dark blue color indicates significant (*p* < 0.001) clusters of low zero-dose children (non-risky areas). Hence, western, eastern and northern parts of Somali and western and central parts of Afar regions were identified to have high zero-dose children (hotspot areas) followed by the Northeastern part of Amhara and southeastern part of Oromia regions. On the other hand, SNNP, Sidama, and the Eastern part of Southwest Ethiopia Peoples regions were identified as cold spot areas for zero-dose children (non-risky areas) (Figure 2).

### 3.4 Interpolation of zero-dose children

**Figure 3:**
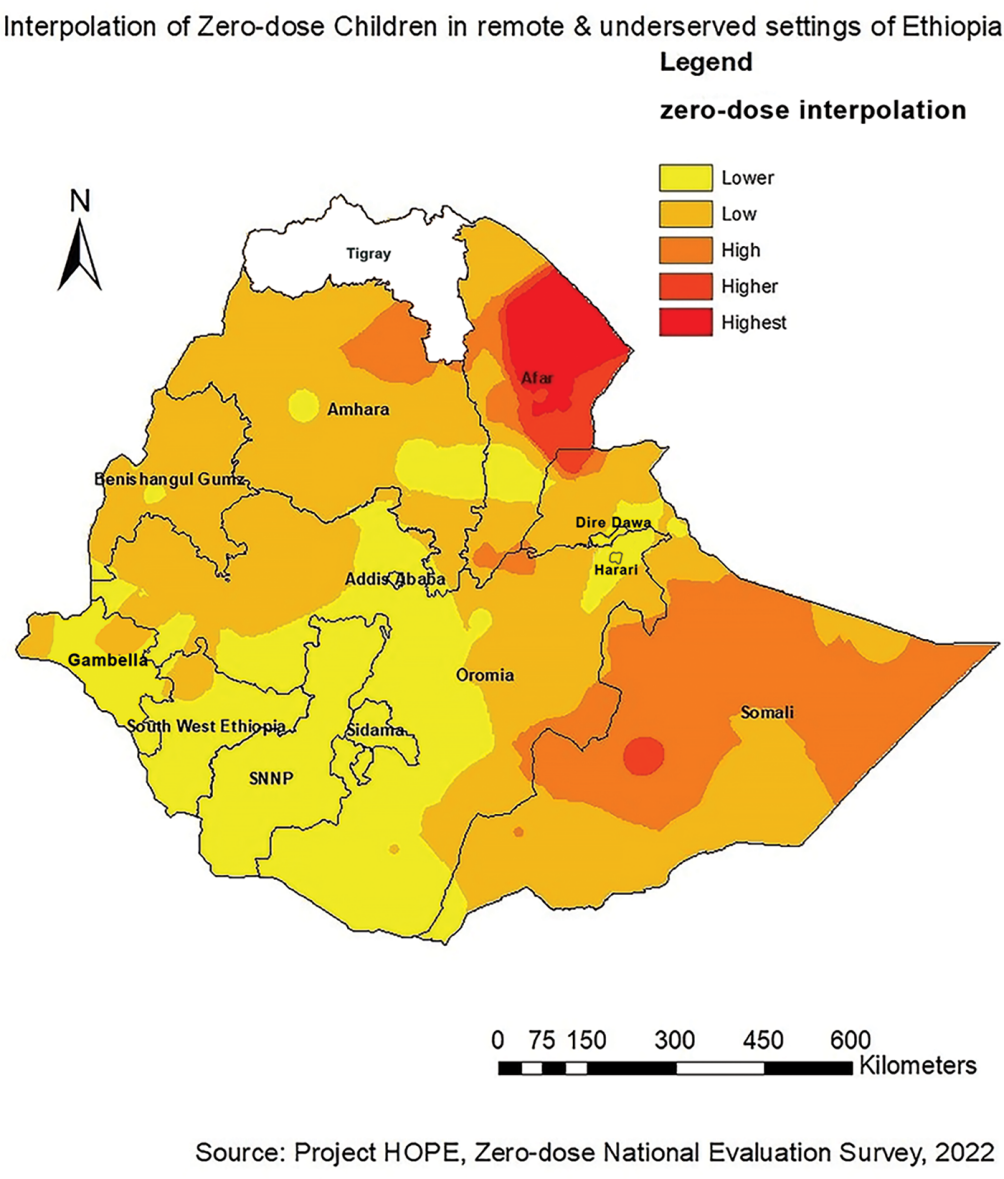
Interpolation of zero-dose children in Ethiopia, a cross-sectional evaluation survey.

Western and central parts of Afar and western, eastern and northern parts of Somali regions were identified as riskier areas for zero-dose children followed by the Northeastern part of Amhara and southeastern part of Oromia regions. However, Addis Ababa, Dire Dawa, Harari, SNNP, Sidama, Southwest Ethiopia Peoples, and parts of Oromia were found to be low-risk areas for zero-dose children (Figure 3).

## 4 Discussion

To our knowledge, this is the first study to map the spatial distribution of zero-dose children in Ethiopia. The study aimed to explore the spatial pattern of zero-dose children aged 12–35 months in Ethiopia. According to this study, a spatial variation of zero-dose children was observed across the study areas. The spatial autocorrelation analysis revealed that the spatial distribution of zero-dose children in Ethiopia was non-random (Global Moran's *I* = 0.178971, *p* < 0.001). Hotspot analysis of the data showed that high zero-dose children (hotspot areas) were observed in western and central parts of Afar and western, eastern and northern parts of Somali regions. The spatial interpolation analysis corresponded with the hotspot analysis results where western and central parts of Afar and western, eastern and northern parts of Somali regions were identified as high-risk areas for zero-dose children. Conversely, cold spot areas were observed in Addis Ababa, Dire Dawa, Harari, SNNP, Sidama, and the Eastern part of Southwest Ethiopia Peoples.

Our study's findings about the spatial distribution of zero-dose children in Ethiopia are consistent with the findings of the 2016 and 2019 EDHS studies which did further analysis on the EDHS datasets and other similar studies conducted in Ethiopia. These surveys and studies showed that the spatial clustering of low immunization coverage was observed in the Eastern and Northeastern parts of the country (12, 16, 27, 32–34). Furthermore, a study done on the geographical variations of immunization defaulting in Ethiopia also showed that most of the hotspot areas (high default value) were located in the eastern (Somali) and northeastern (Afar) parts of the country, whereas most cold spot areas (low default rates) were located in Addis Ababa, Dire Dawa, and Harari (35). Our findings also aligned with the geographical distribution of low childhood measles-containing vaccine coverage where significant clusters were detected primarily in Afar and Somali regions (18, 34).

Explaining the predictors of zero-dose children is as important as mapping their spatial distribution. Predictors of zero-dose children aged 12–35 months in remote and underserved settings of Ethiopia were identified in a separate study. According to this study, living in pastoralist and developing regions was a statistically significant predictor of zero-dose children. This, in turn, was due to mothers'/caregivers' lack of or lower educational status, unavailability of public health facilities, and suboptimal healthcare-seeking behavior (22). This is in alignment with previous studies conducted in pastoralist regions of Ethiopia. According to these studies, the higher prevalence of zero-dose children in Afar and Somali regions may be due to lower health care seeking behaviors, immunization coverage, and vaccine uptake. Moreover, these regions have areas that are hard to reach with nomadic and pastoralist inhabitants who do not have permanent residences (11, 36, 37). The high burden of zero-dose children in Afar and Somali regions might also be ascribed to the existing health care systems that are in the hands of settled populations and rarely have access to nomads due to cultural, political and economic obstacles. Nomadic and pastoralists populations also move seasonally from place to place, which negatively inhibits access to health care services, including immunization (38). Low utilization of health information in these parts of Ethiopia might also contribute to the high burden of zero-dose children (39).

This study also identified the existence of spatial disparities among the different study settings. According to findings of a study that is part of the same cross-sectional survey, these observed disparities in the distribution of zero-dose children in Ethiopia could be attributed to religious beliefs, cultural norms, fear of vaccine side effects and lack of awareness and sustained interventions (40).

Reaching zero-dose children is hampered by a variety of complex obstacles unique to each setting (41). The problem can be addressed by designing-context specific interventions including strengthening horizontal integration of immunization services with other maternal & child health services such as antenatal and postnatal care, sick childcare, nutritional screening, growth monitoring, and family planning; empowering women; strengthening outreach campaigns, supplementary immunization activities (SIAs); Periodic Intensification of Routine Immunization (PIRI); strengthening outreach and static immunization services; providing incentives for health staff; bolstering immunization budget; planning to establish Men Development Army to promote engagement of men in maternal and child health services (20, 21, 40).

This study had several strengths worth mentioning. To the best of our knowledge, this is the first study to map zero-dose children in Ethiopia. Although study settings included areas where there were active conflicts, the study team still managed to collect data in those areas. Ascertainment of child vaccination dropout was based on information gathered from multiple sources. Vaccination cards, medical records, and maternal/caregiver recall were used to ascertain vaccination dropout. This triangulation helped to validate the results and strengthen the quality of the data. Similarly, the use of digital applications and experienced data collectors helped to ensure the gathering of high-quality data. Hotspot areas for zero-dose children were identified using advanced geospatial analyses techniques.

However, the study also had some limitations, including the inability to establish temporal relationships between geospatial covariates and the outcome variable. Another barrier may have been the study's susceptibility to biases, such as nonresponse bias and recall bias, which are directly linked to the cross-sectional design. Mothers and caregivers who did not possess vaccination cards may have forgotten their child's immunization status, which ultimately could have led to misclassification.

## 5 Conclusion and recommendations

The spatial analysis identified that zero-dose children had a significant spatial variation across the study areas. High clusters of zero-dose children were detected in western and central parts of Afar and western, eastern and northern parts of Somali regions followed by the Northeastern part of Amhara and southeastern part of Oromia region.

The findings of our study will be of great importance to guide policymakers in Ethiopia in designing and implementing routine and mop-up vaccination campaigns in the identified hotspot areas to reduce zero-dose children, thereby improving the country's immunization coverage. Moreover, this study might contribute to achieving the 2030 immunization agenda aimed at providing effective and equitable vaccines to everyone, everywhere in the world to realize better health and well-being through avoiding vaccine-preventable diseases.

## Data Availability

The raw data supporting the conclusions of this article will be made available by the authors, without undue reservation.
